# Identification of an exosomal long non-coding RNAs panel for predicting recurrence risk in patients with colorectal cancer

**DOI:** 10.18632/aging.103006

**Published:** 2020-04-04

**Authors:** Yanli Zhang, Hui Liu, Xinfeng Liu, Yulian Guo, Yanlei Wang, Yonggang Dai, Jinhua Zhuo, Bing Wu, Hongchun Wang, Xin Zhang

**Affiliations:** 1 Department of Clinical Laboratory, Qilu Hospital of Shandong University, Jinan 250012, Shandong Province, China; 2 Department of Clinical Laboratory, Shandong Provincial Third Hospital, Jinan 250031, Shandong Province, China; 3 Department of Neurosurgery, Qilu Hospital of Shandong University, Jinan 250012, Shandong Province, China; 4 Department of General Surgery, Qilu Hospital of Shandong University, Jinan 250012, Shandong Province, China

**Keywords:** colorectal cancer, recurrence, long non-coding RNAs, exosomes, biomarker

## Abstract

Recurrence is a major cause of cancer-related deaths in colorectal cancer (CRC) patients, but the current strategies are limited to predict this clinical behavior. Our aim is to develop a recurrence prediction model based on long non-coding RNAs (lncRNAs) in exosomes of serum to improve the prediction accuracy. In discovery phase, 11 lncRNAs were found to be associated with CRC recurrence in tissues using high-throughput lncRNAs microarray and reverse transcription quantitative real-time PCR. And, 9 of them were correlated with their expression levels of serum exosomes. In training phase, a model based on 5-exosomal lncRNAs (exolncRNAs) panel was constructed, and showed high distinguish capability for recurrent CRC patients. ROC showed the panel was superior to serum CEA and CA19-9 in prediction of CRC recurrence. In both training and test sets, high-risk patients defined by the 5-exolncRNAs panel had poor recurrence free and overall survival. And, COX model showed it was an independent factor for CRC prognosis. Moreover, there was a significant relationship in detection of 5-exolncRNAs between plasma samples and paired serum samples. In summary, the 5-exolncRNAs panel robustly stratifies CRC patients’ risk of recurrence, enabling more accurate prediction of prognosis.

## INTRODUCTION

Being the most common gastrointestinal malignancy, colorectal cancer(CRC) contributes to the second leading cause of cancer-related mortality worldwide [1]. Adequate surgical resection is the best therapeutic option for most CRC patients, but approximately 30%-50% of patients undergo curative surgery developed relapse and died of their disease [2]. At present, tumor-node-metastasis (TNM) staging system of American Joint Committee on Cancer (AJCC) has shown limited value for recurrence prediction [3, 4]. Thus, it is urgent to identify factors that are influencing recurrence of CRC, which might promote the prognostic evaluation and individualized treatment.

With the development of transcriptome profiling, long non-coding RNAs (lncRNAs) have been identified and involved in diverse biological processes [5]. LncRNAs are a new class of regulatory RNAs longer than 200 nucleotides with little or no protein-coding ability [6]. They contribute to the gene silencing or activation through various mechanisms, such as epigenetic pathway, chromatin modification, direct interaction with DNAs, RNAs or proteins [7, 8]. In recent years, novel lncRNAs are constantly discovered to act as tumor suppressors or oncogenes involved in the pathogenesis of tumors, including CRC [9–11]. For instance, Tang et al. [12] identified a lncRNA, named glycolysis-associated lncRNA of CRC 1 (GLCC1), which promotes the development of colorectal carcinoma by stabilizing c-Myc protein. A novel long non-coding RNA regulating IL-6 transcription (LNRRIL6) is highly expressed in CRC tissues, and can protect CRC cells via binding to the IL-6 promoter and activating the IL-6/STAT3 pathway [13]. LncRNA nuclear-enriched abundant transcript 1 (NEAT1) has been found as a clinical predictor for CRC recurrence, and functioned as an oncogene to enhance cell proliferation, migration and invasion, and inhibit cell apoptosis by sponging miR-193a-3p [14]. Till date, the lncRNAs associate the recurrent CRC are little known, and few studies about this field only focus on one lncRNA molecular.

Exosomes are nanosized (30-120 nm) vesicles that originate from multivesicular bodies, and secreted from various cells into the extracellular space [15, 16]. It was originally thought to be the cellular “garbage removers”, encapsulating the intracellular "discarded" substance, and has no biological function [17–19]. During the recent years, emerging studies have proven that exosomes are important mediators of cell-cell communication, providing opportunity for exchange of genetic information, and participating in regulation of physiological and pathological processes [20, 21]. When compared with normal cells, tumor cells appear to release more exosomes, which promote tumorigenesis, progression and metastasis through influencing the adjacent or distant cells [22, 23]. Besides enriching the proteins termed “exosomal marker”, such as CD63, CD81, ALIX and TSG101, irrespective of cell type, exosomes released from tumor cells also contain a set of specific molecules mirroring the cells from which they originate [24, 25]. And this provides a noninvasive avenue for searching novel tumor biomarkers.

In this study, we aimed to identify the lncRNAs associated with recurrent CRC by using high-throughput screening, and establish a model based on exosomal lncRNAs (exolncRNAs) panel for effectively predicting CRC recurrence risk and prognosis.

## RESULTS

### Patient characteristics

**Figure 1:**
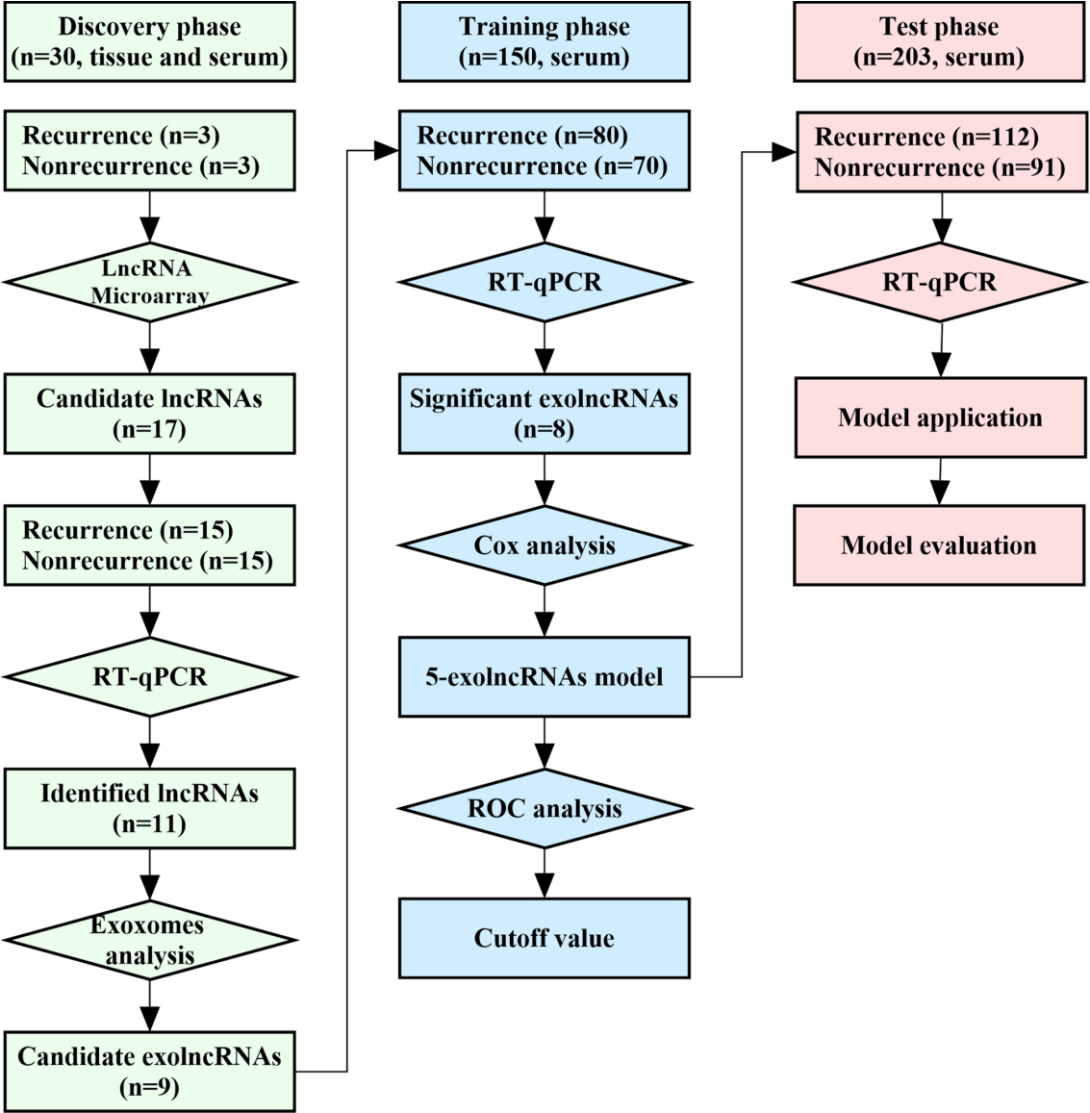
**Workflow of the study.**

This study was mainly designed as 3 phases including a total of 383 CRC patients, and the flowchart was shown in Figure 1. All CRC patients in this study underwent curative resection, and pathologically diagnosed by two experienced pathologists. The postoperative stage was determined according to 2010 AJCC TNM classification. The patients were followed up regularly for up to 5 years. Recurrence free survival (RFS) or overall survival (OS) was defined as the interval between the date of radical surgery and the date of recurrence or death censoring at the time of last contact for survivors. Recurrence patients were those occurred either local or metastatic tumor growth during the follow-up, and patients with RFS more than 5 years were recognized as nonrecurrence. The baseline characteristics of CRC patients were presented in Table 1. There were no significant differences in age, gender, tumor location, tumor size, differentiation, local invasion, and lymph nodes metastasis among CRC patients in discovery set, training set and test set. No significant difference was observed in distant metastasis between a 150-patient training cohort and a 203-patient test cohort. Clinicopathological characteristics were also not significantly different between patients with recurrence and nonrecurrence in the discovery cohort (Supplementary Table 1).

**Table 1:** Clinical characteristics of colorectal cancer patients.

**Parameters**	**Discovery cohort**		**Training cohort**		**Test cohort**		***P* value**
	**No.**	**%**	**No.**	**%**	**No.**	**%**	
Age(year)	61.7±8.7		57.6±12.3		58.8±12.8		0.291^a^
Gender							0.563^b^
Male	17	0.57	69	0.460	98	0.483	
Female	13	0.43	81	0.540	105	0.517	
Tumor location							0.936^b^
Colon	12	0.40	61	0.407	86	0.424	
Rectum	18	0.60	89	0.593	117	0.576	
Tumor size							0.688^b^
<4cm	13	0.43	53	0.353	77	0.379	
≥4cm	17	0.57	97	0.647	126	0.621	
Differentiation							0.107^b^
Well	10	0.33	23	0.153	38	0.187	
Moderate	17	0.57	86	0.573	111	0.547	
Poor	3	0.10	41	0.273	54	0.266	
Local invasion							0.823^b^
T1-T2	9	0.30	44	0.293	54	0.266	
T3-T4	21	0.70	106	0.707	149	0.734	
Lymph nodes metastasis							0.963^b^
No	11	0.37	59	0.393	79	0.389	
Yes	19	0.63	91	0.607	124	0.611	
Distant metastasis						0.000	0.028^b^
No	30	1.00	124	0.827	163	0.803	
Yes	0	0.00	26	0.173	40	0.197	
Recurrence							0.847^b^
No	15	0.50	70	0.467	91	0.448	
Yes	15	0.50	80	0.533	112	0.552	

*P* value^a^ was compared by Kruskal-Wallis test; *P* value^b^ was compared by Chi-square test.

### Identification of recurrence-associated lncRNAs in CRC patients

**Figure 2:**
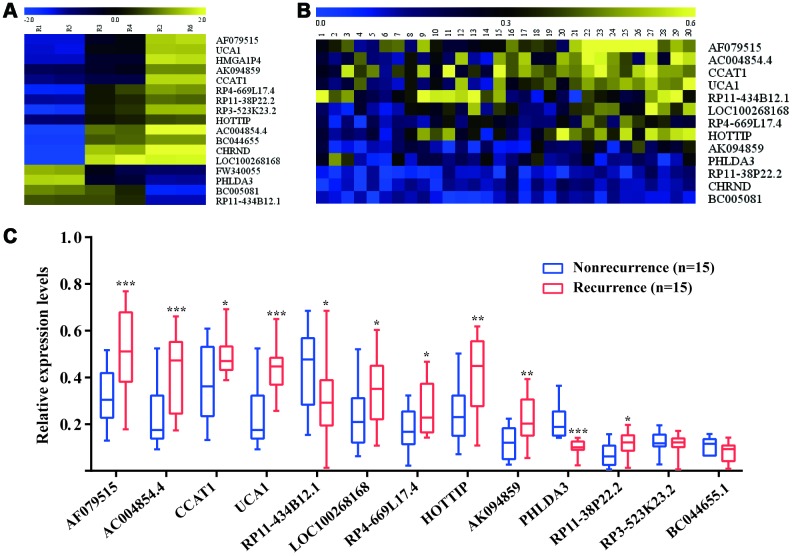
**Identification of recurrence-associated lncRNAs in CRC patients.** (**A**) The heatmap of recurrence-associated lncRNAs identified by high-throughput human genome-wide lncRNA microarray; (**B**) The heatmap of Recurrence-associated lncRNAs detected by RT-qPCR. (**C**) The differentially expressed lncRNAs between recurrence group and nonrecurrence group; ^*^P<0.05, ^**^P<0.01, ^***^P<0.001 [Mann–Whitney U test].

Using high-throughput human genome-wide lncRNA microarray, a total of 4041 lncRNAs were found at least a 2-fold change difference and a *P* value less than 0.05 between tumor tissues and matched adjacent normal tissues (Supplementary Figure 1). The primary data in microarray analysis have been deposited in the Gene Expression Omnibus and the accession numbers is GSE84983. Among them, 17 lncRNAs displayed average expression>10, fold chang >2 and *P* value<0.05 between recurrence and nonrecurrence patients (Figure 2A). Then, the above lncRNAs were detected by RT-qPCR in all CRC patients at discovery phase (Figure 2B). As shown in Figure 2C, significantly differentially expressed 11 lncRNAs were identified, of which 9 upregulated (AF079515, AC004854.4, CCAT1, UCA1, LOC100268168, RP4-669L17.4, HOTTIP, AK094859, and RP11-38P22.2) and 2 downregulated (RP11-434B12.1 and PHLDA3) in CRC patients with recurrence compared to those without recurrence.

### Identification of serum exosomes and levels of recurrence-associated exolncRNAs

**Figure 3:**
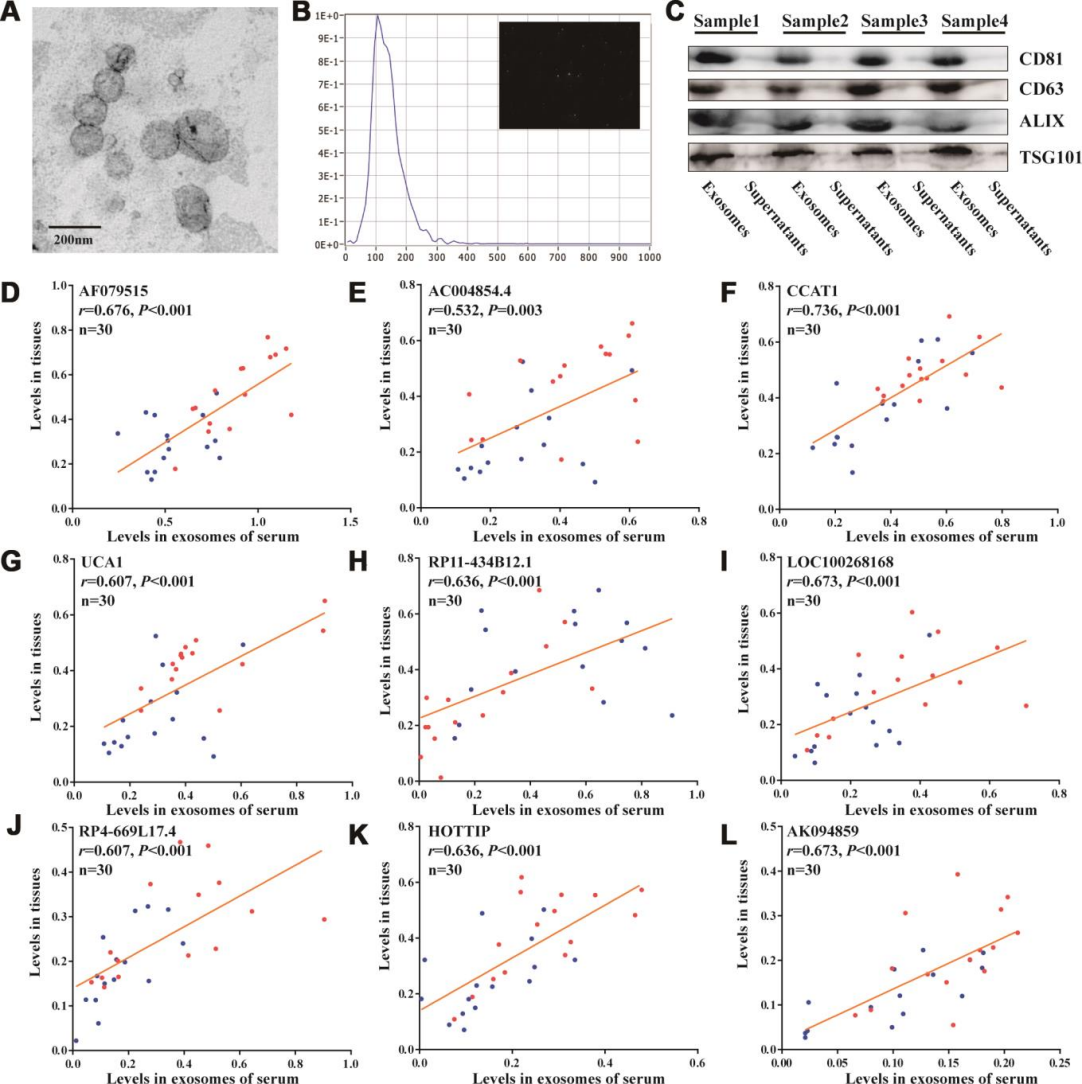
**Detection of recurrence-associated lncRNAs in serum exosomes.** (**A**) Electron microscopy images of exosomes. (**B**) Nanoparticle tracking analysis of the size distributions of exosomes. (**C**) Western blotting analysis of the markers of exosomes (CD81, CD63, ALIX and TSG101). (**D**–**L**) Correlation analyses between each lncRNA expression in tissues and in matched serum exosomes; Red and blue dots represent the lncRNA levels in recurrence and nonrecurrence CRC patients of the discovery cohort, respectively; [Spearman test].

The exosomes extracted from serum exhibited a typical cup-shaped morphology under TEM (Figure 3A). NTA data showed a clear, narrow peak in size distribution at around 90nm, which corresponded to the size of exosomes (Figure 3B). Meanwhile, the exosomes markers (CD81, CD63, ALIX and TSG101) were only expressed in exosomes while not in the supernatants by Western blot (Figure 3C). We tested whether the 11 recurrence-associated lncRNAs could be efficiently amplified in exosomes extracted from corresponding serum. As shown in Figure 3D–3L, the exosomal levels of 9 lncRNAs (AF079515, AC004854.4, CCAT1, UCA1, RP11-434B12.1, LOC100268168, RP4-669L17.4, HOTTIP and AK094859) were significantly correlated with their expression levels of tissues (r>0.5, P<0.05).

**Figure 4:**
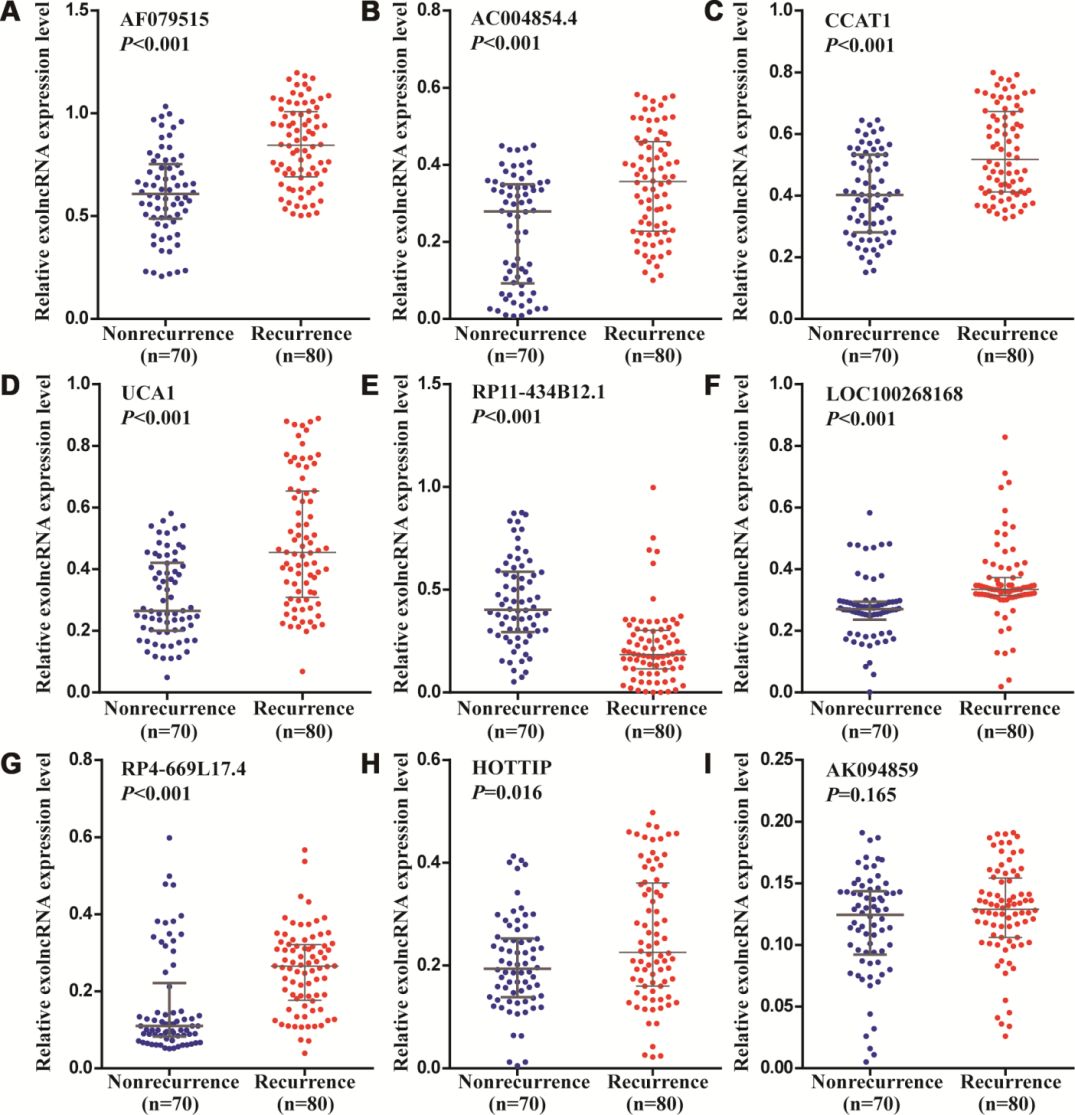
**Relative expression levels of nine exolncRNAs in the training set.** (**A**–**I**) Relative expression levels of (**A**) AF079515, (**B**) AC004854.4, (**C**) CCAT1, (**D**) UCA1, (**E**) RP11-434B12.1, (**F**) LOC100268168, (**G**) RP4-669L17.4, (**H**) HOTTIP, and (**I**) AK094859 in nonrecurrence group (n = 70) and recurrence group (n =80) using RT-qPCR. Data represents the median (interquartile range); [Mann–Whitney U test].

Using RT-qPCR to another 150 CRC patients in training phase, we confirmed the expression pattern of 9 candidates. As shown in Figure 4, the levels of 7 exolncRNAs (AF079515, AC004854.4, CCAT1, UCA1, LOC100268168, RP4-669L17.4 and HOTTIP) were significantly increased, and RP11-434B12.1 was significantly decreased in CRC recurrence group compared with those in nonrecurrence group, while AK094859 levels showed no significant difference between two groups.

### Construction of CRC recurrence prediction model based on exolncRNAs

Univariate Cox regression analysis showed the above 8 recurrence-associated exolncRNAs were significantly associated with RFS. Then, we put them into multivariable cox model, and found only AF079515, CCAT1, UCA1, RP11-434B12.1 and HOTTIP retained significance for RFS. Thus, we derived a formula according to the levels of 5 exolncRNAs, weighted by their regression coefficient, to calculate the risk score: Risk Score=2.924×AF079515+ 2.349×CCAT1+ 2.146×UCA1-1.949×RP11-434B12.1+ 2.475×HOTTIP.

**Figure 5:**
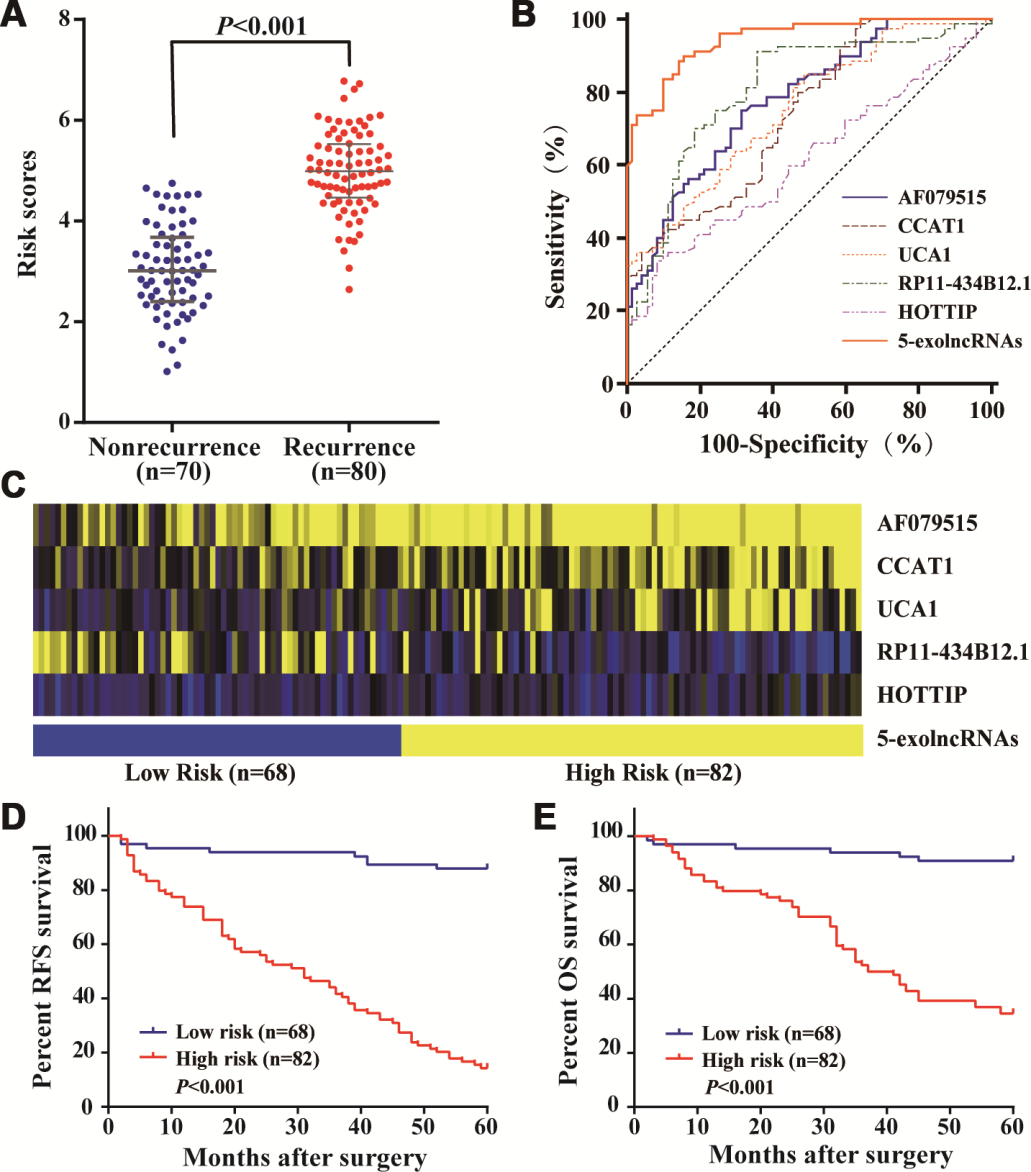
**Construction and performance of exolncRNAs signature in the training set.** (**A**) Risk scores of 5-exolncRNAs were higher in CRC recurrence group than in nonrecurrence group; Data represents the median (interquartile range); [Mann–Whitney U test]. (**B**) ROC curve for discriminating CRC patients with recurrence from those without recurrence based on AF079515, CCAT1, UCA1, RP11-434B12.1 and HOTTIP alone and in combination. (**C**) Heatmap of each lncRNA expressed in CRC patients classified into high- and low-risk groups using 5-exolncRNAs, with yellow indicating higher expression and blue indicating lower expression. (**D**) Kaplan-Meier curves for RFS stratified by 5-exolncRNAs panel in high and low risk using optimal cutoff value (3.998); [log-rank test]. (**E**) Kaplan-Meier curves for OS stratified by 5-exolncRNAs panel in high and low risk using optimal cutoff value (3.998); [log-rank test].

As shown in Figure 5A, the risk scores in CRC recurrence group were significantly higher than those in nonrecurrence group. ROC curve analysis illustrated that 5-exolncRNAs panel could distinguish CRC patients with recurrence from those without recurrence, with the area under the ROC curve (AUC) of 0.891 (95%CI 0.830-0.936), which significantly higher than that for each exolncRNAs detected respectively (Figure 5B). The optimal cutoff value of 5-exolncRNAs panel was 3.998, providing a sensitivity of 88.8% and a specificity of 85.7%. The sensitivity and specificity of each exolncRNA were shown in Supplementary Table 2. Based on the optimal cutoff value (3.998), we divided the CRC patients in the training set into low-risk group with 68 cases and high-risk group with 82 cases (Figure 5C). Kaplan–Meier curve showed patients in the high-risk group were expected to have a dramatically lower RFS rate than those in the low-risk group (Figure 5D). Moreover, the high-risk score patients exhibited shorter OS than patients in the low-risk group (Figure 5E).

### Evaluation of the 5-exolncRNAs panel in the test set

**Figure 6:**
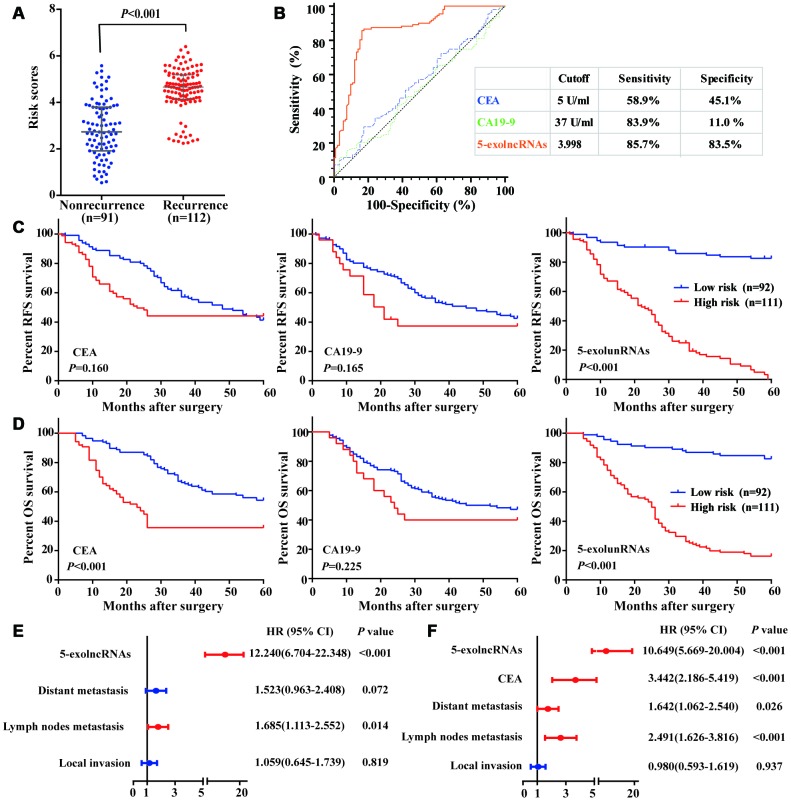
**Evaluation of the 5-exolncRNAs panel in the test set.** (**A**) Risk scores of 5-exolncRNAs were higher in CRC recurrence group than in nonrecurrence group; Data represents the median (interquartile range); [Mann–Whitney U test]. (**B**) ROC curve for discriminating CRC patients with recurrence from those without recurrence based on CEA, CA19-9 and 5-exolncRNAs panel. Sensitivity and specificity are reported. (**C**) Kaplan-Meier curves for RFS based on CEA, CA19-9 and 5-exolncRNAs panel; [log-rank test]. (**D**) Kaplan-Meier curves for OS based on CEA, CA19-9 and 5-exolncRNAs panel; [log-rank test]. (**E** and **F**) Multivariate Cox analysis for RFS (**E**) and OS (**F**) of CRC patients.

We calculated the risk score of 5-exolncRNAs panel according to the formula obtained from the training set in an independent testing set with 203 cases. Similar to the training set, the risk scores in CRC recurrence group were significantly higher than those in nonrecurrence group (Figure 6A). The risk scores of 5-exolncRNAs panel were also significantly in patients with high grade of local invasion, positive regional lymph nodes metastasis, positive distant metastasis and high CEA levels, but showed no relationship with age, gender, Tumor location, Tumor size, Differentiation and CA19-9 levels (Supplementary Table 3). Then, we compared the 5-exolncRNAs panel with serum traditional tumor marker, CEA and CA19-9 in prognosis evaluation. We first employed the ROC analysis to evaluate the predictive accuracy for CRC recurrence, and found 5-exolncRNAs panel had significantly higher AUC value than CEA and CA19-9 (Figure 6B). With their respective cutoff values, 5-exolncRNAs panel showed both high sensitivity and specificity (Figure 6B). According to the Kaplan–Meier curve (Figure 6C and 6D), CRC patients with high high-risk scores exhibited shorter RFS and OS than low-risk patients. Meanwhile, CRC patients with high CEA only showed lower OS (*P*<0.001) while not RFS (*P*>0.05). And CA19-9 had not significant relationship with RFS and OS (both *P*>0.05).

Cox-regression model was used to investigate whether the prognostic value of 5-exolncRNAs panel was independent of other clinicopathological variables. Univariate Cox model analysis revealed a statistically significant association between RFS and local invasion, lymph node metastasis, distant metastasis and 5-exolncRNAs panel, as well as between OS and age, local invasion, lymph node metastasis, distant metastasis, CEA, and 5-exolncRNAs panel. Then, the above factors significantly related to survival were put into the multivariate Cox-regression analysis, and found 5-exolncRNAs panel maintained its significance as independent prognostic factor for RFS and OS (Figure 6E and 6D). The detail data were shown in Supplementary Table 4.

### Evaluation of the 5-exolncRNAs panel in exosomes of plasma samples

**Figure 7:**
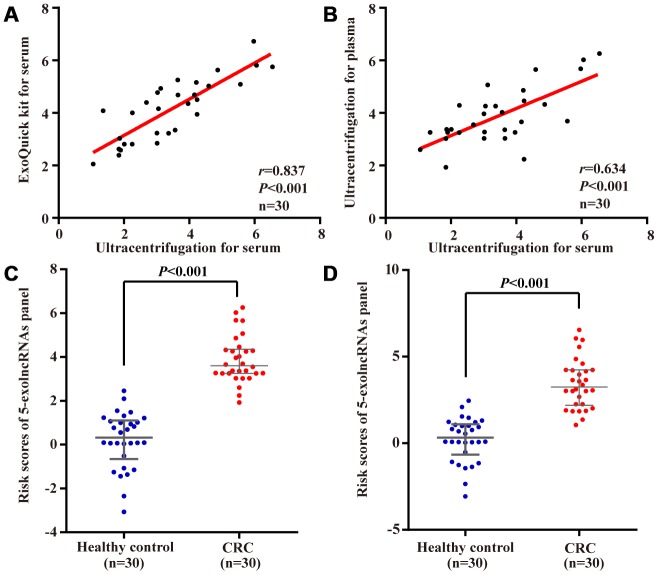
**Evaluation of the 5-exolncRNAs panel in exosomes of plasma samples.** (**A**) The relationship of 5-exolncRNAs panel in serum exosomes isolated from precipitation isolation method with ultracentrifugation method; (**B**) The relationship of 5-exolncRNAs panel in exosomes isolated form plasma and paired serum; (**C** and **D**) Risk scores of 5-exolncRNAs in plasma (**C**) or serum (**D**) of CRC patients were higher than those in healthy controls; Data represents the median (interquartile range); [Mann–Whitney U test].

We first compared ExoQuick isolation method with ultracentrifugation method in detection of 5-exolncRNAs panel using 30 CRC serum samples. As shown in Figure 7A, there was a significant relationship. Using the ultracentrifugation-based method for isolation of exosomes in above 30 CRC patients, we found the levels of 5-exolncRNAs panel in plasma samples were significantly correlated with those in paired serum samples (Figure 7B). The risk scores of 5-exolncRNAs panel in both plasma and serum of CRC patients were significantly higher than those in healthy controls (Figure 7C and 7D).

## DISCUSSION

We have used three-phase study to identify and validate a 5-exolncRNAs panel for prediction of CRC recurrence. Using a predefined formula and cutoff value, the division of CRC patients with and without recurrence was up to 85.7% sensitivity and 87.2% specificity, which superior to the traditional tumor marker, CEA and CA 19-9. Moreover, the patients with high-risk scores of 5-exolncRNAs panel showed short survival in two independent cohorts, which might be an independent factor for prediction of poor prognosis. To our knowledge, it is the first lncRNAs signature identified in exosomes of serum that predicts recurrence in CRC patients.

Recurrence is a leading cause of cancer-related deaths in CRC patients, but the current molecular markers are limited to predict this clinical behavior. In this study, we profiled the lncRNAs using high-throughput technology in CRC patients with and without recurrence. After validation of RT-qPCR, thirteen lncRNAs associated with recurrence were identified, and five of them were used to construct the prediction model. In the signature, UCA1 is putatively oncogenic gene, and correlated with metastasis in various solid tumors [26]. Especially in CRC, increased UCA1 correlated with tumor proliferation and metastasis, and could be used as a predictor for patients’ poor prognosis [27]. UCA1 could also enhance the malignancy and chemotherapeutic resistance of CRC cell lines via sponging the endogenous miR-143/miR-204-5p, which might be one cause of CRC recurrence [28, 29]. CCAT1 is transcribed off the cMYC super-enhancer, and first identified as key activated in initiation and progression of CRC [30]. In CRC, it has been found to function as a ceRNA to antagonize the effects of miR-181b-5p and miR-410 [31, 32]. Ozawa et al. [33] found CCAT1, located within the 8q.24.21 ‘gene desert’, was a superior predictor for tumor recurrence compared to the current clinicopathological indexes in CRC patients. High HOTTIP expression was frequently reported to associate with poor clinical outcomes of cancer patients, including CRC [34]. Down-regulating its expression could inhibit the proliferative activity and metastasis capability of CRC cells by targeting SGK1 [35]. Of note, AF079515 and RP11-434B12.1 in our panel were first found dysregulated in recurrent CRC patients. Although some lncRNAs in the panel have been linked to CRC recurrence previously, their roles were observed in tumor tissues. Recently, some evidences, including ours, suggest tumor related lncRNAs can be selectively packaged into exosomes, and released into human body fluids in a stable form protecting from endogenous RNase [36–38]. And, exosomes are easily extracted from the peripheral blood, providing an alternative non-invasive method for cancer diagnose [39]. Logozzi et al. and Zorrilla et al. have used immune-capture based ELISA, nanosight tracking analysis and nanoscale flow cytometry to detect the plasmatic exosomes, and found that cancer patients have more exosomes in their blood than healthy subjects, indicating exosomes levels might themselves represent a tumor diagnosis marker [40–43]. Moreover, the exosomes carrying the specific tumor biomarkers, such as PSA, provided a more valuable and reliable method than the conventional PSA test [42, 43]. Recently, Cappello et al. [44] have suggested highly specific markers can improve the application of exosomes. Thus, we tested whether the lncRNAs that we identified in tissues could be detected in exosomes of serum. Among them, nine lncRNAs showed concordant expression between tissues and serum exosomes. Interestingly, HOTTIP derived from circulating exosomes recently published by our group can be used as a potential diagnostic and prognostic biomarker of gastric cancer [38]. Therefore, levels of exolncRNAs in serum might be more suitable for preoperative assessment of recurrence risk in real practice.

Furthermore, because of the tissue-, disease-specific expression patterns, lncRNAs hold strong promise as diagnostic and prognostic biomarkers [45]. There are recent reports of lncRNAs signature acting as potential biomarkers in various kinds of malignancies. For example, lncRNA profile study revealed a 24-lncRNA panel associated with the prognosis of patients with gastric cancer, independent of lymph node ratio and postoperative chemotherapy [46]. Li et al. [47] identified a 5-lncRNA signature to improve recurrence prediction of breast cancer. Based on a four-lncRNA prognostic model, stage I ovarian cancer patients can be stratified into three discrete classes of relapse risk [48]. In this study, we constructed a 5-exolncRNAs panel, which served as novel candidate biomarker for distinguishing recurrent CRC patients from those without recurrence. Moreover, five lncRNAs in combination are more accurate and better for recurrence evaluation than each lncRNA alone.

Serological markers, CEA and CA19-9 are the current used for postoperative monitoring of CRC by most clinicians in addition to performing CT at intervals [49]. But they cannot provide sufficient evidence that diagnosis of recurrence before symptoms occurrence [50]. In this study, CEA showed about 50% sensitivity and specificity for diagnosis of CRC recurrence at the given cutoff value of kit. Although CA19-9 provided a high sensitivity, but its low specificity limited the application in clinical practice. Thus, we carried out the formula according to the levels of five exolncRNAs to calculate the risk score of CRC patients. When compared with the two traditional serological markers, the model based on a 5-exolncRNAs panel demonstrated both enhanced sensitivity and specificity. Moreover, ROC analysis showed the AUC of 5-exolncRNAs panel was superior to those of CEA and CA19-9 in distinguishing recurrent CRC. Then, we stratified CRC patients into high- and low- risk groups using a predefined cutoff value, and found patients with high-risk scores had short RFS and OS in both training and test sets. Meanwhile, only CEA showed some relationship with OS. Taking a step further, multivariate Cox model analysis realized the risk score of 5-exolncRNAs panel was a potential prognostic factor independent of traditional clinical parameters or staging systems.

In this study, we have used a commercial exosome precipitation solution for exosomes isolation from serum samples. Via the analyses of transmission electron microscopy, NTA and western blot assay, we found the extracted vesicles appeared similar to exosomes isolated using the repeated round of ultracentrifugation that currently considered as the gold standard. We also compared the two isolation techniques in 5-exolncRNAs panel test, and found a significant relationship. Because the precipitation method requires less time (<1h) and low sample volumes (250μl), which can greatly shorten the turn-around time (TAT), it is more feasible in clinical application for fast detection and large sample sizes. Meanwhile, the ultracentrifugation-based isolation technique seems to often suffer from exosome losses, which might cause a medical accident. To explore the wider application, we detected the 5-exolncRNAs panel in plasma samples of CRC patients. We found their levels in plasma samples were significantly correlated with those in paired serum samples, suggesting the test of 5-exolncRNAs panel was also suitable for plasma sample. Furthermore, 5-exolncRNAs panel showed a good effect for distinguishing CRC patients from healthy subjects.

Though the 5-exolncRNA signature is promising, the limitations in this study should be acknowledged. First, this was retrospective study in nature, and prospective large scale cohorts collected from different institutions are needed to confirm the prediction power before it is applied in clinics. Second, we did not observe the serial level changes of exolncRNA before and after operation or chemotherapy in CRC patients. Third, the function of most lncRNAs are not well annotated until now, especially for AF079515 and RP11-434B12.1 in our signature. Further experimental studies on these lncRNAs are needed to provide information about the mechanism behind signature for understanding the recurrence of CRC.

In summary, lncRNAs expression profile is altered in CRC patients with recurrence compared with those without recurrence. Among them, the 5-exolncRNA signature we discovered in exosomes of serum robustly stratify patients’ risk of recurrence and predict the survival. And this may provide rationale for the implementation of intensive follow-up strategy for those at high risk of recurrence.

## MATERIALS AND METHODS

### Study design, patients and sample collection

This study was designed as 3 phases, and the flowchart was shown in Figure 1. In discovery phase, matched tissues and sera samples were collected from 30 CRC patients (15 cases with recurrence and 15 cases with nonrecurrence) at Shandong Provincial Third Hospital between August 2010 and May 2011 to identify recurrence-related exolncRNAs. Among them, 3 recurrence and 3 nonrecurrence patients were selected for LncRNA microarray analysis. Then, all were detected using reverse transcription real-time quantitative polymerase chain reaction (RT-qPCR) to verify the results of microarray. The confirmed lncRNAs were further tested in exosomes of serum, and the closely related were selected for next study. Next, 353 CRC patients enrolled from Qilu Hospital of Shandong University between January 2012 and December 2013 were randomly split into a 150-patient training set and a 203-patient test set. In training phase, exolncRNAs levels were detected using RT-qPCR in serum of 150 CRC patients to further evaluate their clinical value and construct model. In test phase, another 203-patient set was used to evaluate the prognostic value of 5-exolncRNAs panel. CRC patients with incomplete medical records or receiving any anticancer treatment before surgery were not included in this study. In addition, 30 CRC patients and 30 healthy subjects were collected for testing the 5-exolncRNAs panel in plasma samples. This study was approved by the Ethics Committee of Shandong Provincial Third Hospital and Qilu Hospital of Shandong University, and written informed consent was obtained from each patient.

### Sample processing

Tissues samples were washed in Hanks' balanced salt solution, and immediately stored in liquid nitrogen. Serum samples were separated using 2-step centrifugation (1,600g for 10 minutes followed by another centrifugation at 16,000g for 10 minutes) method and stored at -80°C as we previously performed [51].

### Exosomes isolation and identification

Exosomes were isolated from serum using the ExoQuick™ kit (System Biosciences, Mountain View, CA). In brief, 126 μl ExoQuick solution was added to 500ul serum sample, then incubated at 4°C for 30 min. The mixture was centrifuged at 13000 rpm for 2min, removed supernatant and the exosomes pellet was resuspended using 100ul DEPC water for RNA extraction. Exosomes were isolated from 1ml plasma using differential ultracentrifugation, as follows: 1ml plasma were diluted 1: 1 with PBS, then centrifuged at 2,000g for 30min to remove contaminated cells, and centrifuged at 12,000g for 30min to remove cell debris; collected the supernatant to filter through a 0.22μm filter (Millipore Corp., Bedford, MA, USA), then 100,000g ultracentrifuge for 70min; after 1 wash in PBS, the pellet was resuspended in 100ul DEPC water. Exosomes were mounted onto carbon-coated copper grids, and imaged on transmission electron microscopy (JEM-1-11 microscope, Japan). Nanoparticle tracking analysis (NTA) was performed using ZetaView PMX 110 (Particle Metrix, Meerbusch, Germany). Western blot assay was used to detect the markers of exosomes, CD63, CD81, ALIX and TSG101 (1:1,000; Abcam).

### RNA extraction

Total RNA was extracted from tissues using standard TRIzol method (Invitrogen, Carlsbad, CA). Exosomal RNA was isolated using miRNeasy Mini Kit (Qiagen, Valencia, USA) according to the manufacturer’s instructions. The concentration of RNA was measured using Equalbit® RNA HS Assay Kit (Vazyme, Nanjing, China), along with Qubit™ 3.0 fluorometer (Life Technologies, Thermo Fisher Scientific, Waltham, MA, USA), and the quality was assessed by NanoDrop spectrophotometer (Thermo Fisher Scientific, Waltham, MA, USA) and bioanalyzer 2100 (Agilent Technologies, Palo Alto, CA, USA).

### lncRNAs array

Human genome-wide lncRNA microarray (Arraystar Human LncRNA Microarray V2. 0; Agilent Technology, Santa Clara, CA) was used to measure the expression of lncRNAs in 6 pairs of CRC tissues and matched adjacent normal tissues. The value in the microarray was obtained using Agilent Feature Extraction software (version 11.0.1.1). And then, raw data were performed quantile normalization using the GeneSpring GX v11.5.1 software package (Agilent Technologies).

### RT-qPCR

RNA was first treated with DNase I, and then reverse transcribed into complementary DNA (cDNA) using High Capacity cDNA Reverse Transcription Kit (Takara, Dalian, China), and a no-RT assay was performed along with each batch of experiments. qPCR was performed using SYBR Premix Ex Taq^TM^ (Tli RNaseH Plus) (Takara, Dalian, China), with a positive control containing the gene of interest and a negative template control containing all components except the cDNA. Experiments were performed using a GoTaq 2-Step procedure (95°C for 30 s to activate Taq DNA polymerase, followed by 40 cycles of denaturation with 95°C for 5s and 60°C for 34s) and melting curve analysis on CFX-96 Real-Time System (Bio-Rad, USA). Each experiment was performed in triplicate and an average comparative quantification cycle (Cq) was recorded. The Amplification efficiency of each gene was shown in Supplementary Figure 2. The relative expression level of each lncRNA was normalized with reference genes (GAPDH and UBC), and calculated as we previously described [36, 51]. The primers were synthesized by BioSune Biotechnology (Shanghai, China) and are displayed in Supplementary Table 5.

### Carcinoembryonic antigen (CEA) and carbohydrate antigen 19-9 (CA19-9) assay

Levels of CEA and CA19-9 were measured by electrochemiluminescence method on Cobas E601 Analyzer (Roche Diagnostics GmbH, Germany), and the upper limits were defined as 5 ng/ml and 37 U/ml according to the corresponding kits, respectively.

### Statistical analysis

The Mann-Whitney U test was used to compare median lncRNAs levels of recurrence versus non recurrence. The expression correlation between tissue and serum was using Spearman analysis. Cox model was performed to construct exolncRNA panel and evaluate the independent prognostic factors. The recurrence-free and overall survival curves were drawn by Kaplan–Meier method, and compared by log-rank test. The receiver operating characteristic (ROC) curve was calculated to compare the predictive accuracy of each variable using MedCalc 12.2.1 (MedCalc). The optimal cutoff value of 5-exolncRNAs panel was determined according to Youden index (sensitivity+specificity-1). Two-sided P<0.05 was considered statistically significant.

## Supplementary Material

Supplementary Figures

Supplementary Tables
