# Supplementary material for: Identification of an exosomal long non-coding RNAs panel for predicting recurrence risk in patients with colorectal cancer
Source: Aging (Albany NY). 2020 Apr 4;12(7):6067–88. doi: 10.18632/aging.103006 (PMC7185113; doi:10.18632/aging.103006)
Supplement: Supplementary Figures [file aging-12-103006-s001..pdf]

SUPPLEMENTARY FIGURES

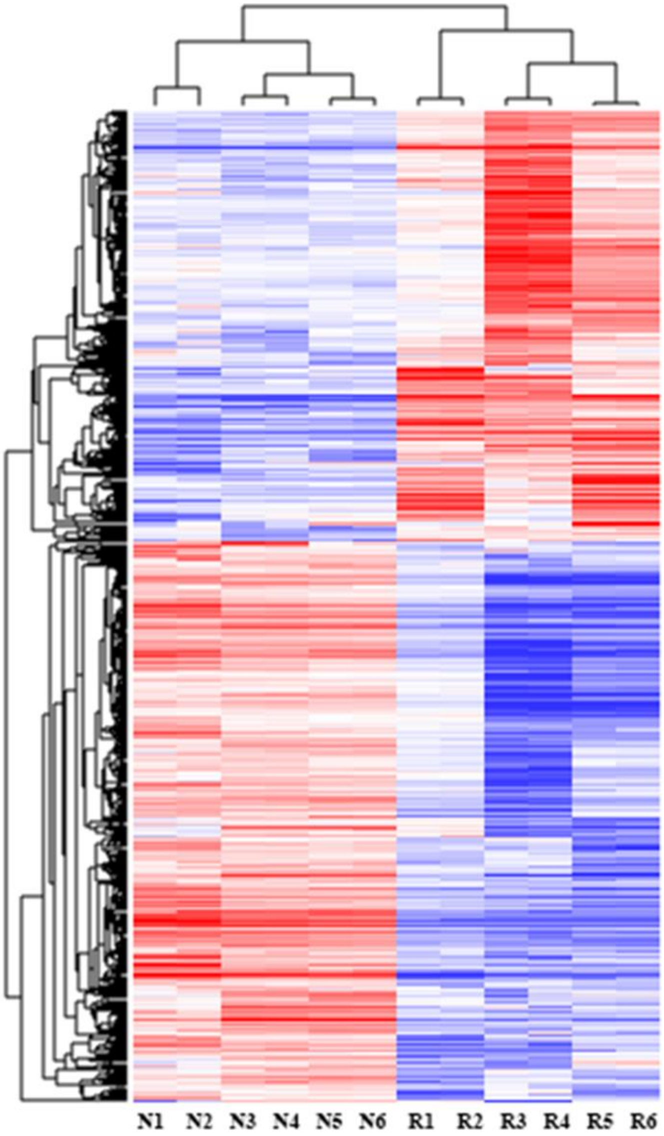

Supplementary Figure 1. Heatmap of different expressed lncRNAs between CRC tumor tissues and matched adjacent normal tissues.

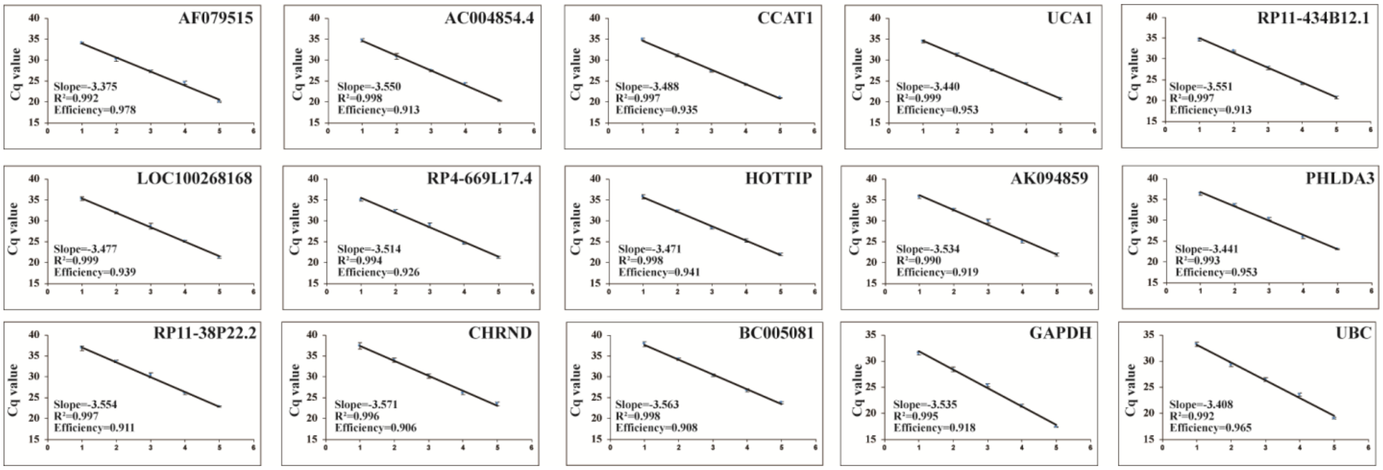

Supplementary Figure 2. Standard curves for lncRNA and reference genes. Cq value is the threshold cycle of qPCR at which fluorescence is detectable. R2 shows the correlation coefficient between Cq value and serial dilutions of sample. Efficiency represents the PCR amplification efficiency.
