# Supplementary material for: Identification of an exosomal long non-coding RNAs panel for predicting recurrence risk in patients with colorectal cancer
Source: Aging (Albany NY). 2020 Apr 4;12(7):6067–88. doi: 10.18632/aging.103006 (PMC7185113; doi:10.18632/aging.103006)
Supplement: Supplementary Tables [file aging-12-103006-s002..pdf]

## SUPPLEMENTARY TABLES

**Supplementary Table 1. Comparison of clinical characteristics between colorectal cancer patients with non-recurrence and recurrence in Discovery cohort.**

| Parameters | Discovery cohort | | | | *P* value |
|---|---|---|---|---|---|
| | Non-recurrence | | Recurrence | | |
| | No. | % | No. | % | |
| Age(year) | 60.7±8.34 | | 62.8±9.16 | | 0.510[a] |
| Gender | | | | | 0.712[b] |
| Male | 9 | 60.00 | 8 | 53.33 | |
| Female | 6 | 40.00 | 7 | 46.67 | |
| Tumor location | | | | | 0.456[b] |
| Colon | 5 | 33.33 | 7 | 46.67 | |
| Rectum | 10 | 66.67 | 8 | 53.33 | |
| Tumor size | | | | | 0.712[b] |
| <4cm | 7 | 46.67 | 6 | 40.00 | |
| ≥4cm | 8 | 53.33 | 9 | 60.00 | |
| Differentiation | | | | | 0.532[b] |
| Well | 6 | 40.00 | 4 | 26.67 | |
| Moderate | 7 | 46.67 | 10 | 66.67 | |
| Poor | 2 | 13.33 | 1 | 6.67 | |
| Local invasion | | | | | 0.690[b] |
| T1-T2 | 5 | 33.33 | 4 | 26.67 | |
| T3-T4 | 10 | 66.67 | 11 | 73.33 | |
| Regional lymph nodes metastasis | | | | | 0.256[b] |
| No | 7 | 46.67 | 4 | 26.67 | |
| Yes | 8 | 53.33 | 11 | 73.33 | |

**Supplementary Table 2. The AUC, sensitivity and specificity of each exolncRNA for distinguishing diagnosis of colorectal cancer recurrence.**

| exolncRNA | Cutoff value | AUC(95%IC) | Sensitivity(%) | Specificity(%) |
|---|---|---|---|---|
| AF079515 | 0.688 | 0.780(0.705-0.844) | 75.0 | 68.6 |
| CCAT1 | 0.335 | 0.740(0.662-0.808) | 97.5 | 37.1 |
| UCA1 | 0.265 | 0.754(0.677-0.820) | 85.0 | 51.4 |
| HOTTIP | 0.312 | 0.614(0.531-0.692) | 33.8 | 91.4 |
| RP11-434B12.1 | 0.355 | 0.809(0.737-0.868) | 91.3 | 64.3 |
| 5-exolncRNAs | 3.998 | 0.947(0.898-0.977) | 88.8 | 85.7 |

**Supplementary Table 3. Correlations between the risk scores of 5-exolncRNAs panel and clinicopathological characteristics.**

| Parameters | No. of patients | Risk scores of 5-exolncRNAs panel | *P*-value |
|---|---|---|---|
| Age | | | 0.427[a] |
| <62 | 101 | 4.13(2.62-4.77) | |
| ≥62 (median) | 102 | 4.08(2.63-4.94) | |
| Gender | | | 0.214[a] |
| Male | 98 | 3.992(2.585-4.713) | |
| Female | 105 | 4.207(2.714-4.915) | |
| Tumor location | | | 0.795[a] |
| Colon | 86 | 4.143(2.644-4.700) | |
| Rectum | 117 | 4.070(2.550-4.796) | |
| Tumor size | | | 0.839[a] |
| <4cm | 126 | 4.135(2.513-4.786) | |
| ≥4cm | 77 | 4.081(2.714-4.788) | |
| Differentiation | | | 0.675[b] |
| Well | 38 | 4.351(2.633-4.808) | |
| Moderate | 111 | 4.005(2.528-4.767) | |
| Poor | 54 | 4.206(2.628-4.783) | |
| Local invasion | | | 0.022[a] |
| T1-T2 | 54 | 3.637(2.312-4.525) | |
| T3-T4 | 149 | 4.247(2.713-4.833) | |
| Regional lymph nodes metastasis | | | <0.001[a] |
| No | 124 | 3.637(2.382-4.493) | |
| Yes | 79 | 4.683(3.885-5.262) | |
| Distant metastasis | | | <0.001[a] |
| No | 163 | 3.885(2.469-4.649) | |
| Yes | 40 | 4.811(4.248-5.326) | |
| CEA levels | | | 0.015[a] |
| <5 ng/ml | 116 | 4.054(2.484-4.493) | |
| ≥5 ng/ml | 87 | 4.605(2.730-5.185) | |
| CA19-9 levels | | | 0.292[a] |
| <5 ng/ml | 178 | 4.081(2.612-4.757) | |
| ≥5 ng/ml | 25 | 4.664(2.780-4.876) | |

Note: Risk scores of 5-exolncRNAs panel were presented as median (interquartile range); *P*-value[a] was estimated by Mann–Whitney U test; *P*-value[b] was estimated by Kruskal-Wallis test.

**Supplementary Table 4. Univariate and multivariate Cox regression model analysis of RFS and OS in CRC patients.**

| Parameters | Categories | Univariate analysis | | Multivariate analysis | |
|---|---|---|---|---|---|
| | | HR (95% CI) | *P* value | HR (95% CI) | *P* value |
| RFS | | | | | |
| Gender | Female *VS* Male | 0.814(0.561-1.180) | 0.278 | | |
| Age | <62y *VS* ≥62y | 0.894(0.616-1.297) | 0.556 | | |
| Tumor location | Colon *VS* Rectum | 1.305(0.900-1.892) | 0.160 | | |
| Differentiation | Well *VS* Moderate *VS* Poor | 0.964(0.726-1.279) | 0.798 | | |
| Tumor size | <4cm *VS* ≥4cm | 0.992(0.677-1.455) | 0.969 | | |
| Local invasion | T1-T2 *VS* T3-T4 | 2.072(1.288-3.334) | 0.003 | 1.059(0.645-1.739) | 0.819 |
| Lymph node metastasis | Negative *VS* Positive | 2.823(1.937-4.115) | <0.001 | 1.685(1.113-2.552) | 0.014 |
| Distant metastasis | Negative *VS* Positive | 3.729(2.451-5.675) | <0.001 | 1.523(0.963-2.408) | 0.072 |
| CEA | Negative *VS* Positive | 1.307(0.895-1.908) | 0.166 | | |
| CA19-9 | Negative *VS* Positive | 1.462(0.848-2.522) | 0.172 | | |
| 5-exolncRNAs panel | Low *VS* High | 14.742(8.325-26.104) | <0.001 | 12.240(6.704-22.348) | <0.001 |
| OS | | | | | |
| Gender | Female *VS* Male | 0.708(0.484-1.034) | 0.074 | | |
| Age | <62y *VS* ≥62y | 1.269(0.871-1.848) | 0.215 | | |
| Tumor location | Colon *VS* Rectum | 1.175(0.805-1.714) | 0.403 | | |
| Differentiation | Well *VS* Moderate *VS* Poor | 0.990(0.743-1.319) | 0.945 | | |
| Tumor size | <4cm *VS* ≥4cm | 1.376(0.942-2.008) | 0.099 | | |
| Local invasion | T1-T2 *VS* T3-T4 | 1.884(1.170-3.035) | 0.009 | 0.980(0.593-1.619) | 0.937 |
| Lymph node metastasis | Negative *VS* Positive | 4.440(2.999-6.576) | <0.001 | 2.491(1.626-3.816) | <0.001 |
| Distant metastasis | Negative *VS* Positive | 4.682(3.114-7.040) | <0.001 | 1.642(1.062-2.540) | 0.026 |
| CEA | Negative *VS* Positive | 1.396(0.808-2.409) | <0.001 | 3.442(2.186-5.419) | <0.001 |
| CA19-9 | Negative *VS* Positive | 2.164(1.482-3.160) | 0.231 | | |
| 5-exolncRNAs panel | Low *VS* High | 9.182(5.355-15.743) | <0.001 | 10.649(5.669-20.004) | <0.001 |

**Supplementary Table 5. Primer sequences for real-time PCR.**

| Primer | Sequence | Product length |
|---|---|---|
| AF079515-F | 5'- ATCAACCCTCAACTATCACA -3' | 122 bp |
| AF079515-R | 5'- GCTATGTACGGTAAATGG -3' | |
| AC004854.4-F | 5'- ATTTGGAGGATAACTCCCAGC -3' | 78 bp |
| AC004854.4-R | 5'- TTTTACAACAGCTTCCGGGG -3' | |
| CCAT1-F | 5'- GCCGTGTTAAGCATTGCGAA -3' | 168 bp |
| CCAT1-R | 5'- TCATGTCTCGGCACCTTTCC -3' | |
| UCA1-F | 5'- TGCCAGCCTCAGCTTAATCC -3' | 153 bp |
| UCA1-R | 5'- TCCCTGTTGCTAAGCCGATG -3' | |
| LOC100268168-F | 5'- ACACATCCCCACTCACGTTC -3' | 133 bp |
| LOC100268168-R | 5'- CTTGCCGGAAACAATGTGGG -3' | |
| RP4-669L17.4-F | 5'- AAATGATTCAACAGGAGGAG -3' | 132 bp |
| RP4-669L17.4-R | 5'- TCTTTGTATCGGTGCTCAG -3' | |
| HOTTIP -F | 5'- CCTAAAGCCACGCTTCTTTG -3' | 142 bp |
| HOTTIP -R | 5'- TGCAGGCTGGAGATCCTACT -3' | |
| AK094859-F | 5'- GGGCAGGTGTTATGTTTCTT -3' | 197 bp |
| AK094859-R | 5'- CTGGGCTGGATATTTGGT -3' | |
| RP11-38P22.2-F | 5'- ACATCCATTGTTGGGGCCTT-3' | 70 bp |
| RP11-38P22.2-R | 5'- GATTTCACAGGCTCCTGGCT-3' | |
| CHRND -F | 5'- ATGGTGTGGCTCCCAGAGAT -3' | 93 bp |
| CHRND -R | 5'- GCCGTAGTGGTAGACAAGCA-3' | |
| BC005081-F | 5'- TGACGCCAATCTCTTGGTCC -3' | 72 bp |
| BC005081-R | 5'- TGTCAGGTATTCCCCCACCT -3' | |
| RP11-434B12.1-F | 5'- GGCGTGGTTATGTGGAGTTG -3' | 194 bp |
| RP11-434B12.1-R | 5'- TTCAAACACGGCAAGCCATT -3' | |
| PHLDA3-F | 5'- GAATGGCCTCTGGACTCACC -3' | 137 bp |
| PHLDA3-R | 5'- GAGGTGGGGGAAGAAGTGTG -3' | |
| HMGA1P4-F | 5'- AAGCCCCATCTCATCCTAGC-3' | 78 bp |
| HMGA1P4-R | 5'- AATAAGCACCCCCGCAGATG -3' | |
| RP3-523K23.2-F | 5'- AAGTTGCTGTGTGGAGCAGT -3' | 210 bp |
| RP3-523K23.2-R | 5'- TGTGGGTTCTGGGCTTTGTT -3' | |
| BC044655.1-F | 5'- GGCTGGCCTGGTTATTTCAA -3' | 111 bp |
| BC044655.1-R | 5'- AGGCTCCAAGAAGGCAAGTA -3' | |
| FW340055-F | 5'- CTCCTTTGGCCATTGCAGTC-3' | 110 bp |
| FW340055-R | 5'- ATTCCAAGGTTGGGAGGGAG-3' | |
| GAPDH-F | 5'-TGCACCACCAACTGCTTAGC-3' | 87 bp |
| GAPDH-R | 5'-GGCATGGACTGTGGTCATGAG-3' | |
| UBC-F | 5'-CCGGGATTTGGGTCGCAG-3' | 70 bp |
| UBC-R | 5'-TCACGAAGATCTGCATTGTCAAG-3' | |
